# Accelerated Multisecret Sharing Scheme Using Fast Matrix Spectral Factorization

**DOI:** 10.3390/e28040369

**Published:** 2026-03-25

**Authors:** Selda Çalkavur, Patrick Solé, Lasha Ephremidze

**Affiliations:** 1 Department of Mathematics, Kocaeli University, 41380 Kocaeli, Turkey; selda.calkavur@kocaeli.edu.tr; 2 I2M (CNRS, Universirty Aix Marseille, Centrale Marseille), 13009 Marseille, France; 3 School of Mathematics, Kutaisi International University, 4600 Kutaisi, Georgia; lasha.ephremidze@kiu.edu.ge

**Keywords:** multisecret sharing, paraunitary matrices, matrix spectral factorization, fast algorithms, finite fields, cryptographic security, secret reconstruction, large-scale distributed systems

## Abstract

In this paper, we propose a novel multisecret sharing (MSS) scheme that integrates a recently developed exponential-speedup matrix spectral factorization algorithm into the construction of paraunitary matrices over finite fields. By exploiting the block-matrix generalization of the Janashia-Lagvilava method, we significantly enhance the efficiency and scalability of the MSS scheme. The proposed method ensures perfect secrecy, collusion resistance, and efficient reconstruction, while enabling practical deployment in large-scale distributed systems such as secure cloud storage, IoT networks, and blockchain authentication. Security and performance analyses demonstrate the superiority of the new approach over existing MSS schemes.

## 1. Introduction

Secret sharing remains a fundamental cryptographic primitive that enables secure and distributed information management. Since its inception by Shamir [1] and Blakley [2] in 1979, secret sharing has evolved to underpin critical technologies such as cloud security [3], distributed ledger systems [4], and privacy-preserving machine learning [5].

With the growing complexity of data systems, multisecret sharing (MSS)—the simultaneous secure distribution of multiple secrets—has gained significant attention. MSS offers storage efficiency and operational scalability, which are vital for applications like federated learning [6] and IoT data aggregation [7].

Multisecret sharing schemes have been studied extensively in the literature, with various algebraic constructions proposed to improve efficiency and flexibility [8]. Classical approaches often rely on polynomial interpolation or linear algebra techniques. More recently, coding-theoretic constructions have also been investigated. For instance, a verifiable multisecret sharing scheme based on LCD quadratic residue codes has been proposed [9], where the share generation process relies on the algebraic structure of linear codes and enables verification of distributed shares. Such approaches demonstrate how coding theory can be used to enhance the reliability and security of MSS schemes.

Recent advancements have addressed various aspects of MSS, including access structure flexibility [10], verifiability [11], and quantum resilience [12]. Particularly notable are MSS schemes incorporating linear complementary dual (LCD) codes [13] and lattice-based constructions [14].

Parallel to these developments, the use of matrix-based methods—especially paraunitary matrices and orthogonal matrix structures—has been explored to enhance the security and reconstruction efficiency of MSS schemes [15,16]. Orthogonal matrices over finite fields ensure invertibility, efficient recovery, and strong resistance against collusion attacks.

However, a major challenge persists: the computational complexity of constructing large paraunitary matrices. Classical approaches to matrix spectral factorization (MSF), such as Wiener–Masani theory [17] and traditional iterative methods [18], are computationally prohibitive for high-dimensional matrices. Even the significant breakthrough by Janashia and Lagvilava [19] in the 2010s, while reducing the complexity of MSF, still involved sequential processing that scales poorly with dimension.

A crucial turning point came with the recent work of Ephremidze et al. [20] in 2025, who introduced an exponential-speedup algorithm for matrix spectral factorization. By extending the Janashia–Lagvilava method to block-matrix formulations and non-commutative polynomial coefficients, they enabled a dramatic reduction in processing time—achieving real-time factorization even for matrices of size 1024 × 1024. Moreover, their approach naturally supports parallel processing, a key requirement for modern cloud and edge computing environments. This work draws direct inspiration from the fast matrix spectral factorization algorithm developed in [20]. We propose a novel multisecret sharing scheme that leverages this significant computational efficiency improvement to efficiently construct large paraunitary matrices for MSS applications.

It is important to note that the proposed scheme induces a block-structured access model. Each secret block is associated with a corresponding block of participants who can jointly reconstruct that secret. While this may resemble parallel executions of secret sharing schemes, the proposed method differs in that all secrets are encoded simultaneously through a single algebraic transformation based on fast matrix spectral factorization. As a result, the shares are globally coupled and generated through a unified encoding procedure, which characterizes the scheme as a block-oriented multisecret sharing framework rather than a collection of independent secret sharing instances.

Our contributions can be summarized as follows:

We integrate fast block-matrix spectral factorization into secret sharing, achieving scalable and efficient construction of the encoding structure. We enhance security guarantees including perfect secrecy, collusion resistance, and non-leakage under finite-field operations. We demonstrate that our scheme scales to large numbers of participants and secrets, enabling practical deployment in systems such as secure federated learning, decentralized IoT networks, and multi-layer blockchain architectures.

The remainder of this paper is organized as follows. Section 2 provides the necessary mathematical background, including definitions of paraunitary matrices, orthogonal transformations over finite fields, and the matrix spectral factorization process. Section 3 surveys related work and contrasts prior MSS approaches with our spectral-factorization-based design. In Section 4, we present a detailed construction of the proposed scheme. Section 5 offers a concrete numerical example to illustrate the encoding and decoding process. Section 6 explores access structures and threshold properties. Section 7 analyzes coalition behavior and the complexity of access verification. Section 8 presents a detailed security analysis, including information-theoretic guarantees and collusion resistance. Finally, Section 9 concludes the paper and discusses potential directions for future work.

## 2. Preliminaries

This section outlines essential mathematical tools and concepts required for the construction and analysis of our proposed multisecret sharing (MSS) scheme. We review paraunitary matrices, orthogonal matrices over finite fields, matrix spectral factorization, and multisecret sharing schemes—each serving a critical role in our design.

Although classical spectral factorization theory is often formulated for analytic matrix functions defined on the unit circle and involving positive-definite structures, in this work we employ an algebraic version of these ideas. In particular, the factorization is applied to polynomial matrices over the finite field $F_{q}$. The references to analytic concepts are used only to motivate the structure of paraunitary matrices and the corresponding factorization framework. The actual construction of the proposed multisecret sharing scheme is performed entirely within the algebraic setting of polynomial matrices over $F_{q}$.

### 2.1. Paraunitary Matrices over Finite Fields

A matrix polynomial U(z) over a finite field $F_{q}$ is called paraunitary if it satisfies the relation

$$U\left(z\right)\tilde{U}\left(z\right)=\tilde{U}\left(z\right)U\left(z\right)=I,$$

where $\tilde{U}\left(z\right)=U^{T}\left(z^{-1}\right)$. Paraunitary matrices are extensively used in signal processing and coding theory due to their energy-preserving and invertibility properties [21]. In the context of secret sharing, paraunitary matrices serve as robust encoding mechanisms. Their algebraic structure guarantees lossless reconstruction and orthogonality among shares, ensuring perfect secrecy and collusion resistance [22].

### 2.2. Orthogonal Matrices over Finite Fields

An orthogonal matrix $O\in {\left(F_{q}\right)}^{n\times n}$ satisfies $O^{T}O=I$, where $O^{T}$ is the transpose. These matrices preserve vector norms and are bijective, making them ideal for applications requiring linear independence and error-free invertibility.

In recent MSS constructions, orthogonal matrices have been used to generate linearly independent share vectors over finite fields. Such designs improve efficiency and enhance resistance to structure-based attacks. For instance, Çalkavur, and Solé [22] developed multisecret sharing schemes over finite fields using orthogonal transformations, enhancing efficiency and resistance to structure-based attacks.

### 2.3. Matrix Spectral Factorization

Matrix spectral factorization (MSF) refers to the decomposition of a positive-definite matrix function S(z) as

$$S\left(z\right)=S_{+}\left(z\right)S_{+}^{H}\left(z\right),$$

where $S_{+}\left(z\right)$ is analytic and invertible in the unit disk. Classical methods, such as those introduced by Wiener and Masani [17], are computationally demanding for high-dimensional matrices. A significant advancement was introduced by Janashia and Lagvilava [19], who developed a novel LU-type MSF method for matrix-valued functions. More recently, Wang et al. [20] extended this method to block matrices, achieving exponential speedup in computation, making it suitable for cryptographic contexts involving large paraunitary matrix constructions.

### 2.4. Multisecret Sharing Schemes

A multisecret sharing (MSS) scheme allows a dealer to share multiple secrets $S_{1},\ldots ,S_{k}$ among participants such that different subsets are authorized to reconstruct different secrets. Each secret may be protected by its own access structure, denoted as $\Gamma_{i}\subseteq 2^{P}$, where *P* is the set of participants.

MSS offers substantial advantages over classical secret sharing, including reduced communication cost, better storage utilization, and compatibility with dynamic access structures. However, achieving efficient and secure MSS in large-scale environments remains a challenge.

Recent work has addressed these limitations through various constructions. For example, Çalkavur, and Solé [22] constructed MSS schemes over finite fields using Blakley’s geometric model, providing flexibility and efficiency in secure data distribution.

## 3. Proposed Scheme Based on Block Paraunitary Spectral Factorization

In order to unify the notation used throughout the paper, we represent the collection of secrets as a global secret vector *S*. This vector is partitioned into several blocks corresponding to different participant groups. Each block represents a subset of secrets associated with a particular participant block. Thus, the block-wise reconstruction procedure can be interpreted as recovering a corresponding component of the global secret vector.

In this section, we describe a multisecret sharing scheme that closely follows the formal structure of our previous work but replaces the random orthogonal matrix construction with the efficient and scalable paraunitary block-matrix factorization method introduced by Wang et al. [20]. Our goal is to preserve the deterministic algebraic integrity of the original scheme while extending it to higher dimensions with better numerical properties and faster generation time.

**Notation and System Model**

To avoid ambiguity, we clarify the notation used throughout the paper. Let *n* denote the total number of participants in the system. The participants are partitioned into *m* disjoint blocks, each containing *M* participants. Hence,

n=m·M.

The dealer distributes a vector of *k* secrets. In the proposed block-based construction, we consider k=M, meaning that each block of participants is associated with one secret block. Thus, the system simultaneously distributes multiple secrets while organizing participants into structured blocks.

More precisely, the set of participants is denoted by

$$P=\{P_{1},P_{2},\ldots ,P_{n}\}.$$

These participants are partitioned into blocks

$$B_{1},B_{2},\ldots ,B_{m}$$

such that each block $B_{i}$ contains *M* participants. The reconstruction of the corresponding secret block requires the cooperation of the participants belonging to the same block.

### 3.1. Setup

Let $F_{q}$ be a finite field of odd characteristic. Let N∈N denote the degree bound for polynomial terms and M∈N the size of blocks in the matrices. Let n=m·M denote the total number of participants, with m≥2 and each participant receiving a share block of length *M*. The scheme distributes a vector of *k* secrets, and in our construction we set k=M, $s={\left(s_{1},\ldots ,s_{M}\right)}^{T}\in {\left(F_{q}\right)}^{M}$.

### 3.2. Construction of the Structured Matrix F(z)

Let $\zeta_{j}\left(z\right)\in F_{q}{\left[z^{-1}\right]}^{M\times M}$ be matrix polynomials of negative degree only, i.e.,

$$\zeta_{j}\left(z\right)=\sum_{n=1}^{N}\Gamma_{j,n}z^{-n},j=1,2,\ldots ,m-1,$$

where $\Gamma_{j,n}\in {\left(F_{q}\right)}^{M\times M}.$ Let $f\left(z\right)=\sum_{n=0}^{N}F_{n}z^{n}\in {\left(F_{q}\left[z\right]\right)}^{M\times M}$ be a polynomial with invertible constant coefficient $F_{0}$, i.e., $det(F_{0})\neq 0.$

Define the block lower-triangular matrix $F\left(z\right)\in F_{q}{\left[z,z^{-1}\right]}^{n\times n}$ as:

$$F\left(z\right)=\left(\begin{array}{cccc} I_{M} & 0 & \ldots & 0\\ 0 & I_{M} & \ldots & 0\\ \vdots & \vdots & \ddots & \vdots \\ \zeta_{1}\left(z\right) & \zeta_{2}\left(z\right) & \ldots & f(z) \end{array}\right)\cdot$$

This matrix serves as a structured block polynomial matrix.

### 3.3. Constructing Paraunitary Matrix U(z)

There exists a paraunitary matrix $U\left(z\right)\in {\left(F_{q}\left[z\right]\right)}^{n\times n}$ of the form

$$U\left(z\right)=\left(\begin{array}{ccc} u_{11}\left(z\right) & \ldots & u_{1m}\left(z\right)\\ \vdots & \vdots & \vdots \\ u_{m-1,1}\left(z\right) & \ldots & u_{m-1,m}\left(z\right)\\ \tilde{u_{m1}}\left(z\right) & \ldots & \tilde{u_{mm}}\left(z\right) \end{array}\right),$$

where $u_{ij}\left(z\right)\in {\left(F_{q}\left[z\right]\right)}^{M\times M}$, 1≤i,j≤m, and $\tilde{u}\left(z\right)=u\left(z^{-1}\right)$. The matrix U(z) satisfies $U\left(z\right)\tilde{U}\left(z\right)=I_{n}$ for all $z\in F_{q}^{*}.$ This matrix can be computed using Wang et al.’s block Janashia–Lagvilava spectral factorization algorithm, which efficiently converts F(z) into $S_{+}\left(z\right)=F\left(z\right)U\left(z\right)$.

### 3.4. Secret Embedding

We encode the secrets $s\in {\left(F_{q}\right)}^{M}$ into the first *M* components of the vector

$$X\left(z\right)=\left(\begin{array}{c} s\\ 0\\ \vdots \\ 0 \end{array}\right)\in {\left(F_{q}\right)}^{n}.$$

Then we compute the shares via the matrix-vector product

y(z)=U(z)·x(z),

where

$$y\left(z\right)=\left(\begin{array}{c} y_{1}\left(z\right)\\ \vdots \\ y_{m}\left(z\right) \end{array}\right),$$

with each block $y_{i}\left(z\right)\in {\left(F_{q}\left[z\right]\right)}^{M}.$

### 3.5. Share Distribution

Each participant $P_{i}$ receives the polynomial block $y_{i}\left(z\right),$ represented by its coefficients in ${\left(F_{q}\right)}^{M(N+1)}$. The total number of nonzero coefficients per share is at most N+1, bounded by the degree of U(z).

### 3.6. Reconstruction

To recover the secret, participants compute the inverse

$$x\left(z\right)=\tilde{U}\left(z\right)\cdot y\left(z\right)\cdot$$

The first block of x(z), which is of size *M*, corresponds exactly to the original secret vector *s*, due to the paraunitary property

$$U\left(z\right)\tilde{U}\left(z\right)=I_{n}.$$

Thus, perfect reconstruction is guaranteed if all shares are received without corruption.

**Example** **1.** *Let us establish a concrete multisecret sharing instance over the finite field*$F_{7}.$ We take the following:

- *Number of blocks: m=2.*
- *Block size: M=1 (i.e., scalar case).*
- *Total number of participants: n=m·M=2.*
- *Maximum polynomial degree: N=1.*

*So, each share is a polynomial of degree of at most 1 in z, with coefficients in* $F_{7}.$

**Step 1: Define a Structured Matrix** F(z)

Following Wang et al. [20], we construct a structured matrix of the form

$$F\left(z\right)=\left(\begin{array}{cc} 1 & 0\\ \zeta (z) & f(z) \end{array}\right),$$

with $\zeta \left(z\right)=6z^{-1},f\left(z\right)=1+2z.$ So

$$F\left(z\right)=\left(\begin{array}{cc} 1 & 0\\ 6z^{-1} & 1+2z \end{array}\right)\cdot$$

This defines a valid positive-definite matrix function $S\left(z\right)=F\left(z\right)F^{H}\left(z\right)$ on the unit circle suitable for spectral factorization.

**Step 2: Compute the Paraunitary Matrix** U(z)

As shown in [20], the paraunitary matrix U(z) can be computed to satisfy

$$S_{+}\left(z\right)=F\left(z\right)U\left(z\right)$$

with

$$U\left(z\right)=\left(\begin{array}{cc} a(z) & b(z)\\ -\tilde{b}\left(z\right) & \tilde{a}\left(z\right) \end{array}\right)\cdot$$

For this simple case, it can be computed that

$$U\left(z\right)=\left(\begin{array}{cc} 2 & 5z\\ 2z^{-1} & 2 \end{array}\right),$$

where the latter matrix belongs to $F_{7}{\left[z,z^{-1}\right]}^{2\times 2}$.

Then the para-conjugate transpose is

$$\tilde{U}\left(z\right)=\left(\begin{array}{cc} 2 & 2z\\ 5z^{-1} & 2 \end{array}\right)\cdot$$

We can verify that

$$U\left(z\right)\tilde{U}\left(z\right)=\left(\begin{array}{cc} 2 & 5z\\ 2z^{-1} & 2 \end{array}\right)\left(\begin{array}{cc} 2 & 2z\\ 5z^{-1} & 2 \end{array}\right)=I,$$

if we perform operations on coefficients in $F_{7}$.

**Step 3: Embed a Secret**

Let the secret be $s=4\in F_{7}$. Form the secret vector

$$x\left(z\right)=\left(\begin{array}{c} 4\\ 0 \end{array}\right)\cdot$$

compute the encoded share vector

$$y\left(z\right)=U\left(z\right)\cdot x\left(z\right)=\left(\begin{array}{cc} 2 & 5z\\ 2z^{-1} & 2 \end{array}\right)\cdot \left(\begin{array}{c} 4\\ 0 \end{array}\right)=\left(\begin{array}{c} 1\\ z^{-1} \end{array}\right).$$

So

Participant 1 receives: $y_{1}\left(z\right)=1;$
Participant 2 receives: $y_{2}\left(z\right)=z^{-1}.$

**Secret Reconstruction**

The participants reconstruct the secret by applying

$$x\left(z\right)=\tilde{U}\left(z\right)\cdot y\left(z\right)=\left(\begin{array}{cc} 2 & 2z\\ 5z^{-1} & 2 \end{array}\right)\left(\begin{array}{c} 1\\ z^{-1} \end{array}\right)=\left(\begin{array}{c} 4\\ 0 \end{array}\right),$$

which is the original secret because of the perfect reconstruction property.

**Conclusion:**

This example shows:

How to construct F(z) and derive U(z), how secrets are encoded via U(z)·x(z), how recovery works via $\tilde{U}\left(z\right)\cdot y\left(z\right)$, and how the paraunitary property ensures perfect reconstruction.

**Theorem** **1.** *(Minimal Access Structure of the Proposed Scheme): Let S be a multisecret sharing scheme constructed using a paraunitary matrix $U\left(z\right)\in F_{q}{\left[z,z^{-1}\right]}^{M\times M},$ applied block-wise to a vector of m secrets $x=(x_{1}^{T},\ldots ,x_{m}^{T})$, where each $x_{i}\in F_{q}^{M}$, and n=m·M total shares are distributed to n participants. Then, the minimal authorized subsets for recovering secret block $x_{i}$ are exactly the sets of any M participants that hold the M shares derived from the i-th block.*

**Proof.**  We prove the theorem in two parts: (1) Sufficiency and (2) Necessity.

**1.**  **Sufficiency:** Let $y_{i}\left(z\right)=U\left(z\right)\cdot x_{i}\in F_{q}{\left[z,z^{-1}\right]}^{M}$ be the vector of shares corresponding to the i<4-th secret block.
  - The matrix U(z) is paraunitary, meaning it satisfies
    $$U\left(z\right)\cdot \tilde{U}\left(z\right)=I_{M}.$$
  - Therefore, $x_{i}$ can be exactly recovered using
    $$x_{i}=\tilde{U}\left(z\right)\cdot y_{i}\left(z\right)\cdot$$
  - Since all *M* components of $y_{i}\left(z\right)$ are required for this matrix-vector multiplication, any group of *M* participants holding those specific shares can fully recover $x_{i}$.
  Thus, these sets are authorized.
**2.**  **Necessity:** Suppose only t<M shares from the *i*-th block are available. Then the reconstruction system becomes
  $$y_{i}^{\left(t\right)}\left(z\right)=U^{\left(t\right)}\left(z\right)\cdot x_{i},$$
  where $U^{\left(t\right)}\left(z\right)\in F_{q}{\left[z,z^{-1}\right]}^{t\times M}$ is a submatrix of U(z).

Since t<M, this is an underdetermined linear system. Therefore, multiple (even infinite) solutions $x_{i}$ exist, and unique reconstruction is impossible. Furthermore, since the scheme is block-wise, shares from other blocks (i.e., $y_{j}\left(z\right),j\neq i$) are encoded independently and do not contain any information about $x_{i}$, due to the orthogonality of the scheme and the structure of the block-wise encoding.

Hence, no subset smaller than *M* participants from block *i*, nor any combination involving shares from other blocks, can reconstruct $x_{i}$.   □

Each secret block $x_{i}$ requires exactly its corresponding *M* shares for reconstruction. These sets are minimal: removing any share prevents reconstruction. Therefore, they form the minimal authorized subsets in the access structure.

- **Threshold Property of the Scheme:** The proposed MSS scheme does not implement a classical (t,n)-threshold scheme for any single secret, but rather a block-wise threshold structure, where each secret block has its own threshold t=M.
- **Explanation:**

In a traditional (t,n)-threshold scheme, any *t* out of *n* participants can reconstruct the secret. In this scheme, only the specific subset of *M* shares corresponding to the same secret block can reconstruct that block. Participants outside a block do not contribute to reconstructing that block, and combining shares across blocks does not help in recovering any one secret.

Thus, each secret $x_{i}$ is protected by an independent threshold structure with threshold *M*, but there is no global threshold *t* valid across all secrets and participants.

**Proposition** **1.** *The proposed multisecret sharing scheme is a block-wise (M,M)-threshold scheme: for each secret block $x_{i}\in F_{q}^{M},$ any subset of exactly M shares corresponding to that block is sufficient to reconstruct $x_{i}$, while any strict subset of fewer than M shares reveals no information about $x_{i}$.*

**Proof.**  Let us fix the notation and structure as described in the scheme:

- A sequence of *m* secrets $x_{1},\ldots ,x_{m}$, each of length *M*, is encoded independently using the same paraunitary matrix $U\left(z\right)\in F_{q}{\left[z,z^{-1}\right]}^{M\times M}$.
- The encoding for each secret block $x_{i}\in F_{q}^{M}$ is
  $$y_{i}\left(z\right)=U\left(z\right)\cdot x_{i}.$$
- The resulting vector $y_{i}\left(z\right)$ is split among *M* participants, one share per participant, so that each participant holds a single component $y_{i,j}\left(z\right)$ for j=1,…,M.

**(1)**  **Sufficiency of *M* Shares**
  To reconstruct $x_{i}$, we need to solve the equation
  $$y_{i}\left(z\right)=U\left(z\right)\cdot x_{i}.$$
  Since U(z) is paraunitary, it is invertible (i.e., $U{\left(z\right)}^{-1}=U^{*}\left(z^{-1}\right)$), and full knowledge of all *M* components of $y_{i}\left(z\right)$ allows us to compute
  $$x_{i}=U^{*}\left(z^{-1}\right)\cdot y_{i}\left(z\right)\cdot$$
  Therefore, the set of all *M* participants that received shares from block *i* is sufficient to recover $x_{i}.$
**(2)**  **Insufficiency of Fewer Than *M* Shares**
  Now suppose a coalition of t<M participants has access to only a subset $y_{i}^{\left(t\right)}$ of $y_{i}\left(z\right)$, consisting of components
  $$y_{i}^{\left(t\right)}\left(z\right)=U^{\left(t\right)}\left(z\right)\cdot x_{i},$$
  where $U^{\left(t\right)}\left(z\right)\in F_{q}{\left[z,z^{-1}\right]}^{t}\times M$ is a submatrix of U(z) formed of *t* rows. Because t<M, the system is underdetermined, and there are infinitely many possible $x_{i}$ consistent with $y_{i}^{\left(t\right)}\left(z\right)$. Moreover, if the secret $x_{i}$ is chosen uniformly at random from $F_{q}^{M}$, then the adversary’s knowledge of $y_{i}^{\left(t\right)}\left(z\right)$ does not reduce the entropy of $x_{i}$, so the mutual information is zero
  $$I(x_{i},y_{i}^{\left(t\right)}\left(z\right))=0.$$
  This confirms perfect secrecy: no partial subset of shares from block *i* reveals any information about $x_{i}$.

□

Each block in the scheme behaves as an independent (M,M)-threshold scheme:

Any *M* shares for block *i* allow recovery of $x_{i}$, while fewer than *M* shares give no information about $x_{i}$. Thus, the proposed scheme has the block-wise threshold property as claimed.

**Corollary** **1.** *This scheme exhibits a structured access policy rather than a uniform threshold, where the minimal access sets are*

$$\Gamma_{i}=\left\{A\subseteq P|A=\left\{P_{i-1}M+1,\ldots ,P_{iM}\right\}\right\}$$

*for each secret block $x_{i},i=1,\ldots ,m.$*

## 4. Statistics on Coalitions

The analysis of coalitions—i.e., subsets of participants attempting to reconstruct one or more secrets—is essential for evaluating the security and efficiency of any multisecret sharing (MSS) scheme. In our proposed scheme, due to the block-wise paraunitary encoding structure, the coalition behavior exhibits predictable and quantifiable patterns.

### 4.1. Authorized Coalitions per Secret

For each secret block $x_{i}$, the minimal authorized set $A_{i}$ consists of exactly *M* participants holding shares derived from that block. Therefore, the number of minimal authorized coalitions per block is exactly one:

#(minimal authorized coalitions per $x_{i})\;=\;1$. The number of total authorized coalitions (i.e., supersets of $A_{i}$) is

$$2^{M}-1$$

since any non-empty superset of $A_{i}$ is also authorized to reconstruct $x_{i}$. The total number of authorized coalitions for all *m* blocks, assuming independence, is:

$$\sum_{i=1}^{m}\left(2^{M}-1\right)=m\left(2^{M}-1\right).$$

### 4.2. Unauthorized Coalitions

A coalition C⊂{1,…,n} is unauthorized for block $x_{i}$ if it contains fewer than *M* participants from $A_{i}$. Given that blocks are distributed to disjoint groups, unauthorized coalitions may contain shares from multiple blocks, but cannot reconstruct any individual block unless they gather all *M* shares for that block. Let t<M. The number of unauthorized subsets of size *t* within each block is

Mt,0≤t<M.

Summing over all such *t*, we get the number of strictly unauthorized subsets per block

∑t=0M−1Mt=2M−MM=2M−1.

Thus, the count of strictly unauthorized coalitions is equal to that of authorized ones—but they differ in structure and security implications.

### 4.3. Implications for Collusion Resistance

Since each block must be fully reconstructed with exactly *M* shares, no coalition of fewer than *M* participants per block can recover any part of the corresponding secret. Moreover, coalitions spanning multiple blocks cannot improve their capability unless they satisfy the threshold for at least one individual block. Therefore, the global security of the scheme scales linearly with the number of blocks *m*, while each block remains independently protected.

This structure provides fine-grained access control and allows for localized compromise analysis. It is particularly well-suited for distributed applications such as cloud storage, federated databases, and multi-party computation systems, where trust boundaries are block-specific rather than global.

### 4.4. Complexity Analysis of Coalitional Structures

In a multisecret sharing scheme with *m* secret blocks and n=m·M total participants, we are interested in analyzing the total number of possible coalitions, the computational complexity of identifying authorized subsets, and the implications for scalability and security.

**(a)**  **Total Number of Coalitions**
  Let $P=\{P_{1},\ldots ,P_{n}\}$ be the set of all participants. The total number of possible coalitions is
  $$|C_{all}|=2^{n}-1$$
  excluding the empty set. This exponential growth is expected in secret sharing settings and motivates efficient structural analysis.
**(b)**  **Authorized Coalitions**
  Recall that secrets are grouped into *m* independent blocks, each with a unique minimal authorized subset of size *M*. For each block $x_{i}$, the number of supersets of the minimal authorized set $A_{i}\subseteq P$ is
  $$|C_{auth}^{\left(i\right)}|=2^{n-(n-M)}-1.$$
  Thus, across all *m* blocks,
  $$|C_{auth}|=m.\left(2^{M}-1\right)\cdot$$
  This is linear in *m* and exponential in *M*, indicating that the block size *M* is the dominant factor in the growth of authorized coalition space.
**(c)**  **Unauthorized Coalitions**
  Unauthorized coalitions consist of all subsets that do not fully cover any minimal authorized block. Since each participant belongs to exactly one block, any coalition missing at least one member from every block’s authorized set cannot reconstruct any secret.
  Let $C_{unauth}$ be the set of all such coalitions. The worst-case number of unauthorized coalitions is
  $$|C_{unauth}|=2^{n}-1-m\left(2^{M}-1\right)\cdot$$
  This shows that as *M* increases, the fraction of unauthorized coalitions dominates, which enhances security—only a small, structured subset of the total coalition space can access secrets.
  The complexity analysis presented in this work is primarily theoretical and is based on the algebraic properties of the proposed matrix spectral factorization framework. The analysis demonstrates the asymptotic computational advantage of the proposed construction compared to conventional approaches.
  While the present work focuses on the theoretical design and complexity analysis, a full-scale optimized implementation over finite fields, including large matrix dimensions such as 1024×1024, remains an important direction for future work. In practice, the efficiency of such implementations depends on several factors, including the choice of finite-field arithmetic libraries and optimized linear algebra routines.
  Developing reproducible benchmark implementations and performance measurements is therefore an interesting topic for further research and will be investigated in future work.
**(d)**  **Complexity of Access Verification**
  To verify whether a coalition C⊆P is authorized, we must partition the participants by block, and check whether any subset contains all *M* shares from one block.
  This process is O(n), as each participant is associated with one block, and at most *m* comparisons are needed. Hence, authorization testing scales linearly in the number of blocks, making it practical for large systems with many secrets.

Table 1 summarizes how the number of total, authorized, and unauthorized coalitions—and the complexity of verifying access—scale with the number of participants *n*, block size *M*, and the number of blocks *m*, highlighting that security grows exponentially with *M* while access verification remains efficient.

## 5. Information-Theoretic Efficiency

The performance of a multisecret sharing (MSS) scheme is not only determined by its security guarantees but also by how efficiently it encodes and distributes information. In this section, we analyze the information-theoretic efficiency of our proposed scheme in terms of rate, idealness, and communication overhead.

### 5.1. Rate and Idealness

Let

- *m* be the number of secret blocks;
- *M* be the size of each block (i.e., the number of secrets per block);
- n=mM be the total number of shares distributed;
- Each share be a vector of polynomials of degree at most *N* thus carrying N+1 field elements per coefficient position.

The total amount of information distributed is

ShareSize=n·(N+1)=m·M·(N+1),

The total size of the secret vector is

SecretSize=m·M

Hence, the rate of the scheme is defined as

$$Rate=\frac{SecretSize}{ShareSize}=\frac{m\cdot M}{m\cdot M\cdot (N+1)}=\frac{1}{N+1}.$$

This shows that the scheme is not ideal when N>0, since the share size exceeds the size of the secrets. However, for small *N*, the overhead remains moderate, and the paraunitary construction ensures high efficiency in large-scale deployments due to its algebraic compactness.

### 5.2. Optimality Under Structural Constraints

While ideal schemes (with rate = 1) are desirable, they are rare when dealing with multiple secrets, structured access policies, or linear algebraic encodings. Our scheme trades ideality for perfect secrecy (unauthorized coalitions gain no information), independent reconstruction per block (scalability), and structured encoding via paraunitary matrices with fast spectral factorization.

This aligns with the design goals of high-performance MSS schemes used in coding-theoretic and signal-processing-inspired settings, where rate-optimality is secondary to flexibility and algebraic security.

### 5.3. Communication Complexity

Each participant receives one polynomial share of degree at most *N*, containing N+1 field elements. Thus, the communication complexity per participant is O(N)·. Moreover, reconstruction requires only the exchange of the *M* polynomial shares corresponding to the target block, making reconstruction complexity linear in block size and constant with respect to total number of participants.

### 5.4. Summary of Efficiency Trade-Offs

Table 2 demonstrates that while the scheme is not ideal in the information-theoretic sense for N>0, it offers a strong balance of security, structure, and scalability, particularly in settings where matrix-based encoding and multisecret functionality are required.

The performance discussion presented in this work is primarily based on theoretical complexity analysis. While the asymptotic results indicate potential efficiency advantages of the proposed matrix-based construction, implementing the scheme and performing experimental benchmarks over large finite fields constitute important directions for future work.

## 6. Security Analysis

In this section, we analyze the security properties of the proposed multisecret sharing (MSS) scheme, with a focus on information-theoretic secrecy, collusion resistance, and resilience against structural and algebraic attacks.

### 6.1. Information-Theoretic Secrecy

Let each secret block $x_{i}\in F_{q}^{M}$ be encoded using a paraunitary matrix $U\left(z\right)\in F_{q}{\left[z,z^{-1}\right]}^{M\times M}$ as

$$y_{i}\left(z\right)=U\left(z\right)\cdot x_{i}.$$

Since U(z) is invertible only as a whole, and each component $y_{i,j}\left(z\right)$ is distributed to a distinct participant, any subset of fewer than *M* shares from block *i* does not suffice to reconstruct $x_{i}$. In fact, such a subset yields a system of underdetermined linear equations, where the entropy of the secret remains unchanged

$$I(x_{i};PartialShares)=0.$$

Hence, the scheme provides perfect secrecy in the information-theoretic sense: unauthorized coalitions learn nothing about the secrets.

### 6.2. Collusion Resistance

For each block, the scheme behaves as an independent (M,M)-threshold scheme:

- Any coalition of fewer than *M* participants from the same block cannot recover the secret;
- Coalitions involving participants from different blocks also fail to reconstruct any secret unless they fully reconstruct a block.

Thus, the scheme is resilient to arbitrary collusions as long as no group collects all shares for a given block.

In the worst-case scenario, an adversary controls up to M−1 participants per block. Since shares from distinct blocks are orthogonal and independently encoded, this provides strong security compartmentalization.

### 6.3. Structural Attack Resistance

The use of paraunitary matrices offers strong resistance to structural attacks:

The encoding matrix U(z) is derived via spectral factorization from a structured Laurent polynomial matrix F(z), whose internal parameters (e.g., $\zeta_{j}\left(z\right),f\left(z\right)$) are assumed to be dealer-private. Even if the adversary knows the general form of U(z), the random coefficients in its polynomial entries prevent reverse engineering unless all shares are known.

Unlike Vandermonde- or Reed–Solomon-based schemes, where matrix structure can be exploited algebraically, paraunitary matrices are nontrivially structured and resistant to linear codeword interpolation attacks.

### 6.4. Algebraic and Linear Reconstruction Attacks

Suppose an adversary attempts to solve

$$y_{i}\left(z\right)=U\left(z\right)\cdot x_{i}$$

by assembling partial rows of $y_{i}\left(z\right)$ and attempting matrix inversion or pseudoinverse reconstruction.

However, when $t<M,U^{\left(t\right)}\left(z\right)\in F_{q}{\left[z\right]}^{t\times M}$ is not invertible, and solving for $x_{i}$ is impossible without leakage. Since all encoding is done over a finite field, brute-force recovery would require testing $q^{M}$ possible values per block, which is computationally infeasible for large *q* or *M*.

Hence, the scheme is resistant to all known linear-algebraic attacks unless a full authorized subset is compromised.

### 6.5. Known-Plaintext and Chosen-Share Attacks

Even if an adversary knows one or more secret–share pairs (e.g., from insider leaks), the security of other blocks remains intact:

Each block uses independent randomness via its own spectral matrix F(z), and there is no cross-block dependency, so compromising block *i* does not help reconstruct block j≠i.

This property ensures forward and backward secrecy across blocks, and robustness against chosen-share attacks where the adversary injects specific input to gain structural information.

Even if an adversary observes shares corresponding to previously known secret vectors, the random parameters used in the share generation process ensure that each execution of the scheme produces statistically independent share distributions. Hence, observing multiple sessions does not help the adversary infer future secrets. As seen in Table 3, the proposed MSS scheme thus achieves strong security guarantees, both in theory and in practice, by combining orthogonality, invertibility, and localized encoding. Its resistance to collusion, structural, and algebraic attacks makes it suitable for adversarial environments where secrets must remain compartmentalized across independent trust domains.

### 6.6. Perfect Secrecy for Unauthorized Sets

Let *S* denote the vector of secrets and let *A* be an unauthorized coalition of participants. The shares distributed in the proposed scheme are generated through linear transformations involving the secret vector and randomly chosen parameters over the underlying finite field. Since the random parameters are chosen independently and uniformly, the resulting share vector observed by any unauthorized coalition corresponds to an undetermined linear system with multiple solutions for the secret vector. More importantly, the randomness ensures that the distribution of the observed shares does not depend on the particular value of the secret.

Consequently, for any unauthorized set *A*, the mutual information between the secret vector and the shares observed by *A* is zero, i.e.,

$$I(S;Shares_{A})=0,$$

which implies that

$$P(S|Shares_{A}=P\left(S\right).$$

Therefore, the proposed scheme satisfies information-theoretic perfect secrecy for all unauthorized coalitions.

**Lemma** **1.** *(Perfect Secrecy) For any unauthorized coalition A, the shares available to A reveal no information about the secret vector S.*

**Proof.**  The shares are generated as linear combinations of the secrets and random coefficients selected uniformly over the finite field. For an unauthorized coalition, the number of equations is strictly less than the number of unknown variables, resulting in an undetermined system. Because the random coefficients are chosen independently and uniformly, every possible secret vector is consistent with the observed shares with equal probability. Hence, the distribution of shares is independent of the secrets, which implies that

$$I(S;Shares_{A})=0.$$

Therefore, the scheme achieves perfect secrecy.    □

**Theorem** **2.** *(Perfect Secrecy for Unauthorized Coalitions) Let S be the multisecret sharing scheme described in this paper, and let $x_{i}\in F_{q}^{M}$ be a secret block encoded as $y_{i}\left(z\right)=U\left(z\right)\cdot x_{i},$ where $U\left(z\right)\in F_{q}{\left[z,z^{-1}\right]}^{M\times M}$ is paraunitary and private to the dealer. Then, any coalition C⊂{1,…,n} that holds fewer than M shares from a given block i gains no information about $x_{i}$. That is,*

$$I(x_{i};{\left\{y_{i,j}\left(z\right)\right\}}_{j\in C})=0$$

*whenever |C|<M.*

**Proof.**  Let C⊂{1,…,M} be a subset of t<M participants who receive partial shares from block $x_{i}$. Let $y_{i}^{\left(t\right)}\left(z\right)\in F_{q}{\left[z,z^{-1}\right]}^{t}$ denote the vector of shares they possess, and $U^{\left(t\right)}\left(z\right)\in F_{q}{\left[z,z^{-1}\right]}^{t\times M}$ be the submatrix of U(z) corresponding to their rows. Then

$$y_{i}^{\left(t\right)}\left(z\right)=U^{\left(t\right)}\left(z\right)\cdot x_{i}.$$

Since t<M, the system of equations is underdetermined. There are $q^{M-t}$ possible secret vectors $x_{i}\in F_{q}^{M}$ consistent with the observed shares $y_{i}^{\left(t\right)}\left(z\right)$. If $x_{i}$ is drawn uniformly at random, then

$$H\left(x_{i}|y_{i}^{\left(t\right)}\left(z\right)\right)=H\left(x_{i}\right)=M\log q\Rightarrow I\left(x_{i};y_{i}^{\left(t\right)}\left(z\right)\right)=0.$$

Thus, the coalition learns nothing about $x_{i}.$ This conclusion holds for every block independently due to the independence of encoding.   □

This theorem confirms that the scheme achieves Shannon-perfect secrecy for any unauthorized subset of participants, even under full knowledge of the encoding structure and polynomial degrees.

**Corollary** **2.** *(Adaptive Security Against Share Corruption) Let an adversary be allowed to adaptively corrupt participants in the proposed MSS scheme, with the goal of learning a secret block $x_{i}\in F_{q}^{M}$. Then, as long as the adversary obtains fewer than M shares from the corresponding block, their advantage remains zero, regardless of the corruption order of strategy. Formally, for any adaptive adversary A, let $C_{t}\subset \left\{1,\ldots ,M\right\}$ be the subset of shares from block i revealed to A after t<M adaptive corruptions. Then*

$$I(x_{i};{\left\{y_{i,j}\left(z\right)\right\}}_{j\in C_{t}})=0,\forall t<M.$$

**Proof.**  The proof follows directly from Theorem 2. At each step t<M, the adversary receives a partial observation

$$y_{i}^{\left(t\right)}\left(z\right)=U^{\left(t\right)}\left(z\right)\cdot x_{i}$$

with $U^{\left(t\right)}\left(z\right)$ of rank at most t<M. Hence the entropy of $x_{i}$ remains unchanged. The adaptivity of the adversary (i.e., choosing the next participant to corrupt based on previously observed shares) does not increase the information gained, because no linear combination of fewer than *M* linearly independent rows of U(z) reveals anything about $x_{i}$ due to underdetermination.

Therefore, the adversary’s information gain is still zero until t=M.   □

### 6.7. Security Model

In order to clarify the security guarantees of the proposed scheme, we explicitly describe the threat model considered in this work.

We assume a semi-honest adversarial model in which participants follow the protocol but may attempt to infer information about the secrets from their available shares. The dealer is assumed to be trusted and responsible when generating the shares. The scheme is analyzed in a single-session information-theoretic setting, where a set of secrets is distributed among participants in a single execution of the protocol.

An adversary may corrupt an arbitrary subset of participants and obtain all shares belonging to that subset. Such a coalition is called unauthorized if it does not satisfy the reconstruction condition of the scheme. The adversary may also choose the secret vector (chosen-secret scenario) or observe shares corresponding to known secrets (known-plaintext scenario). However, because the shares are generated using independent randomness over the underlying finite field, the distribution of shares observed by any unauthorized coalition remains independent of the secret values.

Therefore, the scheme achieves information-theoretic secrecy, meaning that unauthorized coalitions gain no information about the secrets beyond what is implied by the access structure.

## 7. Performance Analysis

In this section, we evaluate the computational and communication performance of the proposed multisecret sharing (MSS) scheme. Our analysis considers the cost of share generation, secret reconstruction, matrix construction, and scalability with respect to the number of secrets, the number of participants, and the degree of the polynomial encoding.

### 7.1. Share Generation Efficiency

Each block of secrets $x_{i}\in F_{q}^{M}$ is encoded via a paraunitary matrix $U\left(z\right)\in F_{q}{\left[z\right]}^{M\times M}$, resulting in shares:

$$y_{i}\left(z\right)=U\left(z\right)\cdot x_{i}$$

The complexity of this multiplication is $O(M^{2}\left(N+1\right))$, where *N* is the degree bound of the polynomials. Since the scheme applies this encoding independently to each of the *m* blocks, the total cost across all secrets is

$$O(mM^{2}\left(N+1\right))\cdot$$

This cost is modest and parallelizable across blocks, enabling practical scalability to large values of *m* (number of secrets).

### 7.2. Matrix Construction via Spectral Factorization

The most computationally intensive component is constructing the paraunitary matrix U(z). We utilize the fast block-matrix spectral factorization algorithm of Wang et al. [20], which has the following features:

It computes a spectral factor U(z) of a given structured matrix F(z) by operating recursively on block rows. The method has complexity of approximately

$$O(M^{2}\log M)$$

for each block, due to recursive sub-block orthogonalization and fast transforms (e.g., Fourier-based projections). Because U(z) is computed once by the dealer and reused for all encoding operations, this one-time cost is amortized over many secrets or sessions.

### 7.3. Secret Reconstruction Cost

Given a full set of *M* shares from block $y_{i}\left(z\right)$, the reconstruction process involves computing:

$$x_{i}=\tilde{U}\left(z\right)\cdot y_{i}\left(z\right)\cdot$$

This is again a matrix-vector multiplication over polynomials of degree ≤ *N*, and the complexity is

$$O(M^{2}\left(N+1\right))\cdot$$

Since only one block is involved in reconstructing a given secret, the cost remains independent of the total number of secrets or participants.

### 7.4. Communication Overhead

Each participant receives a share $y_{i,j}\in F_{q}\left[z\right]$ of degree at most *N*, represented as N+1 field elements. Therefore,

- Per participant communication: O(N+1);
- Total communication: n(N+1)=mM(N+1).

This overhead is linear in both the number of secrets and the polynomial degree *N*, for which a small value is typically chosen for practical implementations.

### 7.5. Scalability and Parallelism

Key features supporting scalability:

- **Block-wise encoding:** Each block can be processed independently, enabling full parallelism across secrets.
- **Matrix reuse:** Once the paraunitary matrix U(z) is computed, it is reused for all blocks and all sessions.
- **Participant scalability:** The number of participants grows linearly with the number of blocks (i.e., n=m·M), without increasing per-block reconstruction cost.

## 8. Comparative Analysis with Zhou and Tang’s Matrix-Based MSS Scheme

To objectively evaluate the strengths of our proposed multisecret sharing (MSS) scheme, we compare it against the matrix projection-based multisecret sharing scheme proposed by Zhou and Tang [23], which is among the earliest to explore matrix-theoretic methods for encoding multiple secrets.

Their approach relies on projecting a secret matrix into a public space using random matrices, and reconstructing secrets by solving matrix equations. In contrast, our scheme uses structured paraunitary matrices derived from spectral factorization, enabling more efficient, modular, and secure sharing of secrets.

### 8.1. Parametric Comparison Table

Let the following notation apply:

- *m*: number of secret blocks;
- *M*: number of secrets per block (block size);
- n=m·M: number of participants;
- *N*: degree of polynomial encoding (proposed scheme only);
- *q*: finite-field size $F_{q}$.

As shown in Table 4, the proposed scheme provides stronger security guarantees and improved parallelism while maintaining similar communication costs.

**Numerical Comparison:**

Assume that

- m=10 blocks;
- M=3 secrets per block;
- N=2;
- q=256.

The practical impact of the parametric differences becomes more evident in the numerical setting provided in Table 5, confirming the scalability and security advantages of our approach.

**Discussion:**
  Advantages of the Proposed Scheme:
  **Structured Local Thresholding:** Unlike Zou, our scheme enforces strict block-level access structures.
  **Perfect Secrecy:** Unauthorized subsets of participants obtain no information, even under adaptive attacks.
  **Paraunitary Matrices:** Algebraically richer and cryptographically safer than ad hoc random matrices.
  **Parallel Encoding:** Independent block-wise encoding and reconstruction scale better for large systems.
  **One-time Matrix Cost:** The paraunitary matrix can be precomputed once and reused across all blocks.

Compared to Zou’s matrix-projection MSS, the proposed scheme

- Achieves stronger security guarantees;
- Maintains consistent communication and computation efficiency;
- Enables highly parallel, modular deployments in practical systems.

These advantages make it especially suitable for large-scale, multi-party environments where confidentiality and efficiency must coexist.

### 8.2. Comparison with Recent Multisecret Sharing Schemes

Recent research on multisecret sharing has explored several algebraic frameworks, including coding-theoretic constructions. For example, a recent verifiable MSS scheme based on LCD quadratic residue codes utilizes the structure of linear codes to distribute multiple secrets while providing share verification capabilities.

In contrast, the scheme proposed in this work adopts a matrix-based framework built upon fast matrix spectral factorization. Instead of relying on coding theoretic properties, the proposed method leverages structured matrix transformations to generate shares and distribute multiple secrets simultaneously among participants. While code-based MSS schemes emphasize verifiability and error-resilient properties inherited from coding theory, the present construction focuses on computational efficiency and structured participant organization through block-based access structures. These two approaches represent complementary directions in the design of modern multisecret sharing schemes.

A comparison between the proposed scheme and a recent code-based multisecret sharing construction is summarized in Table 6. As shown in Table 6, the two approaches rely on different mathematical frameworks. The proposed scheme is based on matrix spectral factorization, whereas the recent scheme utilizes LCD quadratic residue codes from coding theory.

Furthermore, the share generation mechanisms differ significantly. In the proposed approach, shares are generated through structured matrix transformations, enabling simultaneous distribution of multiple secrets. In contrast, the code-based scheme generates shares through linear code encoding, which naturally supports verification mechanisms.

Another key difference lies in the design objectives. The code-based construction emphasizes verifiable secret sharing, allowing participants to check the correctness of distributed shares. On the other hand, the proposed scheme focuses primarily on computational efficiency and structured block-based participant organization. Overall, as illustrated in Table 6, the two approaches represent complementary directions in modern multisecret sharing research: coding-theoretic methods emphasize verification and algebraic robustness, while the proposed matrix-based construction highlights efficient secret distribution through fast matrix operations.

## 9. Conclusions

In this paper, we introduced a novel multisecret sharing (MSS) scheme that leverages paraunitary matrices constructed via fast matrix spectral factorization. The proposed scheme supports the secure, efficient, and modular sharing of multiple secrets by encoding them into independent blocks using structured polynomial transformations. Each block is protected by a strict (M,M)-threshold access structure, and the resulting shares possess strong information-theoretic security: no coalition of fewer than *M* participants can learn any partial information about a given secret.

Our scheme achieves several key advantages over classical approaches:

It supports block-local reconstruction, enabling high parallelism and scalability for systems involving a large number of secrets. The use of paraunitary matrices ensures invertibility and orthogonality while offering enhanced resistance to structural and algebraic attacks. Unlike schemes such as that of Zou, which rely on linear projection with potentially insecure matrix hiding assumptions, our scheme guarantees perfect secrecy against both static and adaptive adversaries. The overall communication complexity remains linear in terms of the number of secrets and polynomial degree, while matrix construction is a one-time cost.

Quantitative analysis further shows that our scheme maintains competitive efficiency compared to established MSS methods, while significantly improving on access control flexibility and coalition resistance. Its strict separation of blocks, predictable complexity, and compatibility with fast algebraic operations make it highly suitable for modern cryptographic applications, including distributed storage, federated systems, and post-quantum secure architectures.

In future work, our framework could be extended to support dynamic secret updates, verifiability, or public parameter settings, and further integrated with lattice-based or code-based cryptographic primitives. Future work will include implementing the proposed scheme over large finite fields and conducting experimental benchmarks to evaluate its performance in practical cryptographic settings.

## Figures and Tables

**Table 1:** Summary.

Property	Value/Complexity
**Total coalitions**	$2^{n}-1$
**Authorized coalitions per block**	$2^{M}-1$
**Total authorized coalitions**	$m2^{M}-1$
**Unauthorized coalitions**	$2^{n}-1-m2^{M}-1$
**Coalition verification complexity**	O(n)
**Scalability**	Linear in *m*, exponential in *M*

**Table 2:** Overview.

Property	Value/Interpretation
**Scheme rate**	$\frac{1}{N+1}$
**Idealness**	Not ideal if N>0
**Perfect secrecy**	Achieved for all unauthorized subsets
**Communication per participant**	O(N)
**Reconstruction cost**	O(M) per block
**Suitability**	Efficient for large *m*, small-to-moderate *N*

**Table 3:** Summary of security guarantees.

Threat Model	Resistance Level
**Unauthorized partial coalitions**	Perfect secrecy (information theoretic)
**Cross-block collusion**	Fully resistant (blocks encoded separately)
**Structural decoding or matrix attacks**	Strongly resistant (via paraunitary design)
**Brute-force over finite fields**	Infeasible if $q^{M}$ is large
**Known-plaintext or insider leaks**	Localized, no leakage to other blocks

**Table 4:** Parametric comparison table.

Feature	Zou [23]	Proposed Scheme
Secret model	Matrix of multiple secrets	Vector of independent secret blocks
Encoding method	Linear projection via random matrices	Paraunitary matrix via spectral factorization
Threshold enforcement	Partial; depends on linear solvability	Strict block-wise threshold (M,M)
Share structure	Matrix columns or vectors	Polynomial vectors of degree ≤ *N*
Share size	*M* elements per secret	N+1 elements per secret
Information rate	Varies, not optimal	$\frac{1}{N+1}$ (predictable and structured)
Reconstruction cost (per block)	$O(M^{3})$ for matrix inversion	$O(M^{2}\left(N+1\right))$
Security against coalitions	Computational (depends on matrix hiding)	Information-theoretic (perfect secrecy)
Resistance to structural attacks	Low–moderate	High (algebraically unpredictable)
Parallelism	Limited	Full block-level parallelism
Matrix generation complexity	Low (random matrices)	Moderate $O(M^{2}\log M)$ (one-time)

**Table 5:** Numerical comparison.

Metric	Zou [23]	Proposed Scheme
Total secrets shared	30	30
Total shares	30	30
Share size (field elements)	3	3
Total communication	30×3=90	30×3=90
Secret-to-share ratio (rate)	≤0.3	$\frac{1}{N+1}=0.33$
Reconstruction per block	O(27)	O(27)
Unauthorized info leakage	Possible (linear leakage)	Zero (perfect secrecy)
Adaptivity resistance	Not addressed	Resists adaptive corruption

**Table 6:** Comparison between the proposed scheme and a recent code-based multisecret sharing scheme.

Feature	Code-Based MSS (QR Codes) [9]	Proposed Scheme
Mathematical basis	LCD quadratic residue codes	Matrix spectral factorization
Secret distribution	Linear code encoding	Matrix transformation
Verification	Verifiable shares	Not explicit
Access structure	Code-defined	Block-based
Main focus	Verifiable secret sharing	Computational efficiency

## Data Availability

Data is contained within the article.
